# Adaptability of water resources development and utilization to social-economy system in Hunan province, China

**DOI:** 10.1038/s41598-023-46678-9

**Published:** 2023-11-09

**Authors:** Yang Lu, Ying Su, Can Cui, Li Ren, Ke Zhang, Yuzhang Wang, Jialiang Yang, Yuequn Huang

**Affiliations:** 1 College of Marxism, Hohai University, Nanjing, Jiangsu 210098 China; 2 College of Hydrology and Water Resources, Hohai University, Nanjing, Jiangsu 210098 China; 3 Hunan Water Resources and Hydropower Survey, Design, Planning and Research Co., Ltd, Changsha, Hunan 410007 China; 4 Hunan Provincial Water Resources Development & Investment Co., Ltd, Changsha, Hunan 410007 China

**Keywords:** Environmental social sciences, Hydrology

## Abstract

The interplay of water resources with social-economy spheres involves a reciprocal feedback mechanism. With the acceleration of the construction process of modernized water networks in Hunan Province, investigating the adaptation status of the "Water-Social-Economy " composite system (WSE) is crucial for promoting sustainability. This study clarifies the connotation of the adaptability of WSE, and the quantitative analyses were conducted through coupling coordinative degree, harmonious development capacity, and the evolution of development lag types among the 14 cities of Hunan Province from 2005 to 2020. The results show that: (1) The development index of the water resources subsystem (WRS) showed a “downward-fluctuation-upward” trend, while the development index of the social-economy subsystem (SES) showed signs of great improvement, the former didn’t catch up with the latter. (2) The coupling coordination degree of WSE developed well, and reached the coordinative development stage by 2020, but the unbalanced spatial pattern between north to south and east to west still exists and is further intensified. (3) The development ability of WSE improved while the harmony ability reduced, and the development rate of WRS and SES hasn’t achieved dynamic synchronization. Finally, the policies and suggestions to improve the adaptability are put forward, which is of instructive significance for the sustainable development of water suitability.

## Introduction

Water resources are a strategic foundational asset that supports high-quality economic development and maintains social security and stability^1^. However, in recent years, the current state of water resource development and utilization in China has not aligned with the demands of economic-social development^2^, severely constraining the efficient utilization of water resources and hindering sustainable and high-quality economic-social development^3^. Therefore, investigating the correlation between water resources and economic-social development and their adaptability holds significant practical implications.

The key to attaining adaptability between water resources and economic-social development lies in achieving a balanced match of various factors such as water resources, economy, and society, and this implies maintaining an ideal state featuring coupling, coordination, and harmonious balance between the status of water resources development and utilization, environmental water conditions, and economic-social development within a certain period and under certain scientific and technological conditions^4^. In existing research, both Chinese and international researchers have conducted qualitative and quantitative studies on adaptability within the water resources-dominated complex system, using various methods from different dimensions. The exploration mainly revolves around the following aspects:

From the perspective of measuring and evaluating the coordinated and sustainable development of water resources, society, and the economy. In 1990, Norgaard^5^ proposed a theory related to coordinated development, which provided a theoretical basis for water resources evaluation. On this basis, in 2007, Wame^6^ proposed to emphasize that the integrated management of water resources should be carried out under the premise of sustainable development of the ecosystem, to maximize the economic and social benefits. Based on the theory of coordination between water resources and social-economy development, many scholars have used mathematical models to evaluate the level of water resources development and utilization in various places. For example, Li et al.^7^ assessed the environmental adaptability of the human-sea economic system in the coastal areas of Liaoning Province between 2000 and 2004, using the entropy-weighted TOPSIS method and a panel Tobit model. Wang et al.^8^ established a coupling coordinative degree model and a spatial autocorrelation analysis model to investigate the coordinated development patterns of resource-environment-economy in 30 Chinese provinces between 2005 and 2019. Bian et al.^9^ researched the adaptability between water resource carrying capacity and social-economy development in the Taihu Lake Basin using the method of development adaptability coordination measurement. Zhang et al.^10^ employed a coupling model to analyze the dynamic evolution of the coordinated development of water resources and the economy in 31 Chinese provinces between 1987 and 2017. Although the evaluation models used are varied, all of these studies tried to measure the degree of matching between the degree of water resources development and social-economy development more objectively.

Over the past century, changes in the climate environment and rapid industrial development have caused fluctuations in the supply and demand of water resources in various regions of the world. For this reason, some scholars have combined the theories of climate change and water demand for agricultural cultivation with water resources evaluation. For example, Lautze et al.^11^ analyzed the mechanisms of interactions between regional water resource allocation, global climate change, and the sustainable development of social-economy. Charlton et al.^12^ studied the impact of climate change on regional water resources, considering England as their study area. Park et al.^13^ considered climate uncertainty and constructed a multistage distributionally robust optimization model to study the robust and sustainable water resource allocation scheme in the Tucson area, guiding the construction of water infrastructure in the region under climate uncertainty. Fu et al.^14^ developed a robust optimal allocation model for agricultural irrigation water by introducing a penalty cost variable in the objective function. This approach facilitated the determination of optimal target values and water allocations for different crops based on distinct water sources. Peiman et al.^15^ developed a robust optimization allocation model for regional management systems, aimed to minimize system costs and reduce distribution cycles to the maximum level. The evaluation of water resources allocation considering the changing environment provides an important reference for the medium- and long-term water resources development and utilization in the evaluation area.

Utilizing mechanistic simulation models such as system dynamics to simulate and predict the relationships within the water resource-dominated complex system is a research hotspot. System dynamics is a mature quantitative methodology for modeling complex feedback interactions in systems. While it offers unique advantages in quantifying the correlations and structures within complex systems, it requires high precision in abstracting and quantifying the relationships among various factors^16^. Naeem et al.^17^ conducted a comprehensive literature review on the application of system dynamics modeling (SDM) in sustainable water supply-demand management (WSDM). They highlighted that sustainable development mechanisms are effective tools for formulating sustainable water supply-demand management strategies. Dawadi et al.^18^ utilized a system dynamics model to analyze the water supply balance among states in the Colorado River Basin in the United States. The study contributes to long-term planning for water resource managers to meet future demand. Hewage et al.^19^ conducted a study using a system dynamics-based approach to establish a comprehensive water-energy-carbon (WEC) relationship model for urban water systems (UWS). The study revealed significant differences in water, energy, and carbon aspects within the UWS when implementing various water- and energy-based intervention measures. In Quaranta et al.’s study^20^, a European Union (EU)-wide assessment was presented to quantify the benefits of urban greening in terms of availability of green water, reduction of cooling costs, and CO_2_ sequestration from the atmosphere, for different climatic scenarios.

In recent years, there has been an increasing trend of exploring the Water-Energy-Food Nexus (W–E–F nexus), with many scholars conducting research on this interconnection from national and basin perspectives. In terms of theoretical research, Sherwood et al.^21^ developed an Environmental Input-Output Life Cycle Assessment (EIO-LCA) model as a means of determining the use of water resources, energy resources, and food resources in various sectors of the U.S. economy. Ziliaskopoulos et al.^22^ proposed a Bi-Level Linear Programming (BLP) methodology for the development of subsidy policies and introduced a generalized model based on W–E–F Nexus relationships. Ioannou et al.^23^ proposed a W–E–F Nexus analysis methodology that allows stakeholders to choose sustainable development goals based on benefit orientation. Regarding the study area, W–E–F Nexus is suitable for evaluating the degree of water resources system matching in different size areas such as countries, basins, and regions. For example, Janez et al.^24^ quantified the correlations as well as uncertainty among W–E–F Nexus systems in 175 countries based on national-level data, and the results showed that W–E–F Nexus indicators are closely related to GDP. Papadopoulou et al.^25^ took Greece as an example, focused on exploring locally oriented indicators and policy in the W–E–F Nexus model. The "Water–Energy–Food (W–E–F Nexus)" nexus relationship has richer considerations and more comprehensive evaluation perspectives than previous evaluation methods and can provide more valuable suggestions for water resources development planning in the evaluation area.

The current research on the adaptability of water resource complex system mainly focuses on the evaluation of water resource carrying capacity, the overall coupling effect of water resource complex system, and the evaluation of water resource security space. The research object mainly focuses on the overall state of the water resource complex system, and the development state of different subsystems has not been detailed, and the accurate definition of "adaptation" is lacking. Insufficient attention has been paid to the research on synergy and competition in the adaptation process between water resources and social-economy complex systems, especially the research on the dynamic evolution of coordinated development between the two from the spatiotemporal dimension, which makes it difficult to clarify the specific mutual feedback relationship between sustainable use of water resources and stable social-economy development at the present stage. In terms of the decomposition degree of influencing factors, most methods do not fully consider the attributes of water endowment, resulting in less decomposition of influencing factors. Regarding the extended application of water resource complex system adaptation models, there is a need to explore and enrich the application of the model from multiple perspectives.

Hunan Province, an important component of China's water network skeleton, formed a radial river network centered on Dongting Lake (the second largest freshwater lake in China), which is a national strategic reserve base of water resources and flood storage field. However, influenced by various factors such as atmospheric circulation and topography, the spatio-temporal distribution of water resources in Hunan Province is highly uneven. For example, Changsha–Zhuzhou–Xiangtan urban agglomeration gathered over 50% of the province's total GDP, and its water resources utilization ratio has approached the upper limit of 40% with an intensified water resources overload phenomenon. Hengyang–Shaoyang–Loudi drought corridor, an important agricultural production base, the irrigation guarantee rate of which fell below 70%. The existing water infrastructure is relatively weak in ensuring water security, the shrinking of rivers, lakes, and ponds leads to poorer water system connectivity, which makes it more difficult to control ecological pollution and excessive heavy metal, resulting in limited development of the Dongting Lake Economic Belt. On the whole, the construction of inter-basin and inter-region water diversion projects is insufficient, and the interconnected and synchronous-asynchronous cooperated water network pattern has not been formed. According to the “14th Five-Year Water Supply Plan of Hunan Province” and the requirements of modern water network construction, it is necessary to adhere to the comprehensive coordination of water resources and social-economy adaptation relationship, so as to guide the development, utilization, conservation, and management of water resources in Hunan Province in the new era.

Compared with the existing research, this paper focuses on the comprehensiveness of evaluation indexes and the diversity of methods, reflecting the connotation of the appropriateness of water resources and social-economy development as much as possible from multiple perspectives and in-depth in the research, to provide theoretical references and decision-making support for the planning of the coordinated and sustainable development of water resources and social-economy development in Hunan Province of China.

## Materials and methods

### Study area overview and data sources

**Figure 1:**
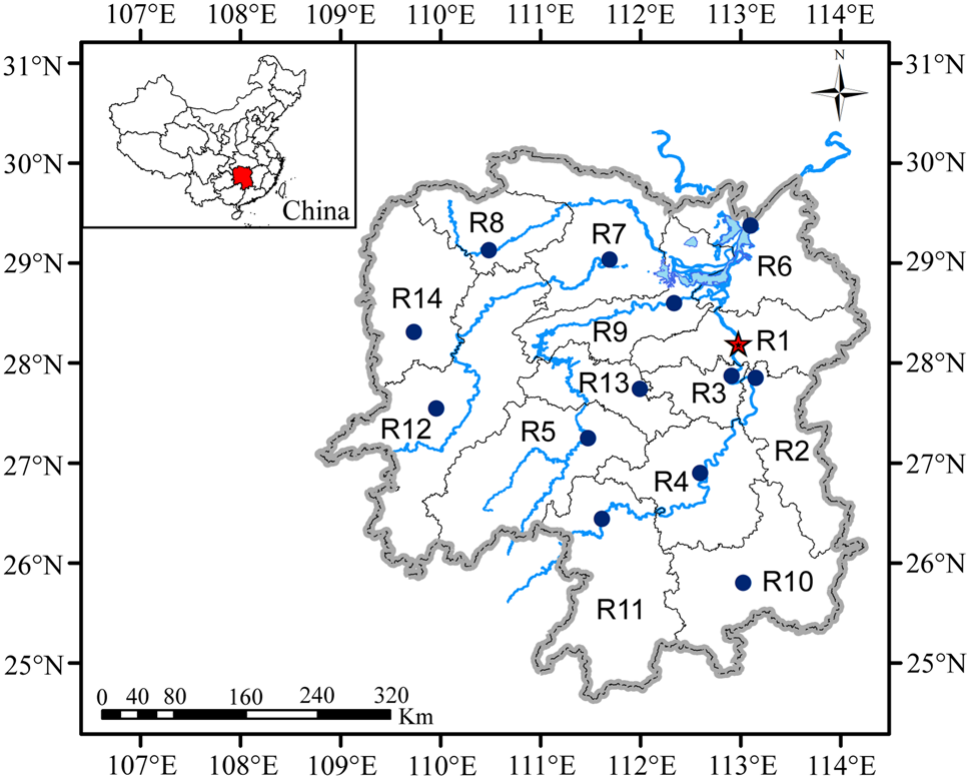
Overview Map of the Study Area. Graphed by ArcGIS 10.2. Maps of Hunan Province and China were downloaded from Resources and Environment Science and Data Center, Institute of Geographic Sciences and Natural Resources Research, CAS (Open access: https://www.resdc.cn/).

Hunan Province, located in central-south China, spans from 24° 38’ N to 30° 08’ N and from 108° 47’ E to 114° 15’ E, and it covers a total area of 211,800 km^2^, which represents 2.2 % of the country's land area. The province comprises 14 administrative divisions (as shown in Fig. 1). These include Changsha City (R1), Zhuzhou City (R2), Xiangtan City (R3), Hengyang City (R4), Shaoyang City (R5), Yueyang City (R6), Changde City (R7), Zhangjiajie City (R8), Yiyang City (R9), Chenzhou City (R10), Yongzhou City (R11), Huaihua City (R12), Loudi City (R13), and Xiangxi Prefecture (R14), as illustrated in Fig. 1. In 2020, the gross domestic product (GDP) of Hunan Province reached 4,178.149 billion yuan. As of May 2021, the province had a resident population of 66.448 million.

Hunan Province has an average annual precipitation of 1454 mm and a total average water resource of 169.5 billion m^3^. The surface water resources amount to 168.8 billion m^3^, whereas the total available water resources are approximately 83.5 billion m^3^. Due to unstable monsoons and topography, precipitation in Hunan Province exhibits spatial and temporal variations. The rainy season typically lasts only three months throughout the year, accounting for 50 % to 60 % of the annual rainfall. The spatial distribution of annual precipitation shows a general decrease from east to west, with larger amounts in the southern, central, and eastern fringes and smaller amounts in the central-western and northern fringes. With the development of the economy and society, domestic water consumption in Hunan Province has been on a continuous upward trajectory since 1991. Industrial water consumption demonstrates an "inverted U-shaped" phenomenon, characterized by an initial upward trend, reaching a peak, and subsequently declining, in line with the changing trend of the Water Kuznets curve^26^ Agricultural water consumption has shown a consistent downward trend since 1991.

The relevant indicator data required for assessing the adaptability of the water resources and social-economy complex system in Hunan Province mainly come from the "Hunan Statistical Yearbook" and the "Hunan Water Resources Bulletin" from 2005 to 2020.

### Construction of the evaluation framework

**Figure 2:**
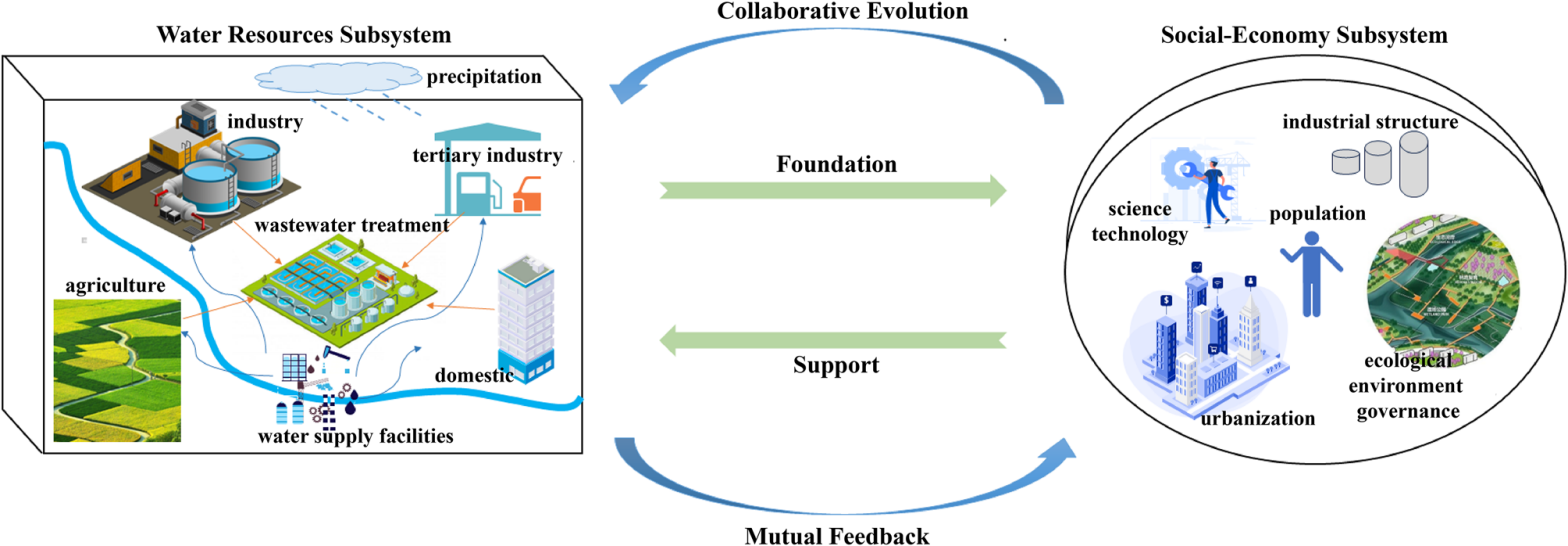
Collaborative evolution and feedback mechanism of the “water-social-economy” composite system.

The adaptability of water resources and social-economy development refers to the ideal state of coupling coordinated development between water resources utilization and social-economy efficiency under certain periods and scientific & technological conditions, and forms a state of mutual adaptation based on supply–demand balance. There is an interactive and interinhibitive feedback mechanism between the “water–social-economy” composite system (WSE). Water resources support or restrict the development of the social-economy, while the latter would have an important impact on the quantity, quality, and utilization efficiency of the former. The collaborative feedback relationship between the two is shown in Fig. 2.

In this study, a multi-level indicator system is established in a top-down manner, consisting of three levels: the objective, criterion, and indicator levels. This system serves as an evaluation framework for the WSE, composed of two parts: the water resources subsystem (WRS) and the social-economy subsystem (SES). The WRS comprises 3 aspects: (1) Natural occurrence conditions, contains the basic carrying capacity, the impact of climate change, and spatial distribution equilibrium of water resources; (2) Development and utilization degree, mainly measured by the surface and underground water supply capacity and availability; (3) Water consumption, which is reflected in the dependence of domestic, production, and ecology on water resources. The SES comprises 2 aspects: (1) Economic development, mainly linked to GDP and its growth rate, industry output and its proportion, and water use efficiency, etc.; (2) Social structure, generally expressed by population, urbanization, cultivated land service conditions, etc. The specific indicators are presented in Table 1^27,28^.

**Table 1:** Regional composite system evaluation indices.

Target layer	Guideline layer	Serial number	Indicator layer	Description and calculation of indicators	Unit	Positive and Negative
Water resources	Occurrence conditions	C_1_	Water resources per capita	Total water resources/total population	m^3^/person	Positive
		C_2_	Annual precipitation	Climate change Impacts	mm	Positive
		C_3_	Water resources per unit administrative area	Total water resources/area	100,000,000 m^3^/km^2^	Positive
	Development degree	C_4_	Surface water availability	Surface water supply capacity	100,000,000 m^3^	Positive
		C_5_	Groundwater availability	Groundwater supply capacity	100,000,000 m^3^	Positive
		C_6_	Water resources development and utilization	Exploited water resources/total water resources	-	Positive
	Water consumption	C_7_	Agricultural water consumption	Dependence of agriculture on water resources	m^3^	Reverse direction
		C_8_	Industrial water consumption	Industrial dependence on water resources	m^3^	Reverse direction
		C_9_	Ecological water consumption	Dependence of the three industries on water resources	m^3^	Positive
		C_10_	Water consumption per capita	Total water consumption/total population	m^3^/person	Reverse direction
Social- economy	Economy	C_11_	GDP per capita	GDP/total population	Yuan	Positive
		C_12_	GDP growth rate	Economic development rate	%	Positive
		C_13_	GDP share of the primary industry	Proportion of primary industry output in GDP	%	Reverse direction
		C_14_	GDP share of secondary production	Proportion of secondary industry output in GDP	%	Reverse direction
		C_15_	GDP share of three industries	Proportion of tertiary industries output to GDP	%	Positive
		C_16_	Water consumption of 10,000 Yuan GDP	Water consumption/GDP	m^3^/10,000 yuan	Reverse direction
		C_17_	Water consumption of 10,000 yuan of industrial-added value	Annual water consumption/industrial value added	m^3^/10,000 yuan	Reverse direction
	Society	C_18_	Population size	Number of people carried	10,000 people	Positive
		C_19_	Population density	Total population/area	10,000 people/km^2^	Reverse direction
		C_20_	Population growth rate	Population dynamic pressure	%	Reverse direction
		C_21_	Urbanization rate	Social development level	%	Positive
		C_22_	Arable land per capita	Total arable land/total population	km^2^/person	Positive

This study adopts the FAHP-Entropy method-based subjective–objective combination weighting method to determine the weights of the 22 indicators mentioned above. This approach combines subjective attitudes with objectivity to ensure a more rational process for determining the weights of the evaluation system indicators. The specific steps for combining weights using FAHP-Entropy are as follows:

1. The FAHP^29^ method is used to weight the indicators. This method utilizes a scale of 0.1 to 0.9 to construct a judgment matrix. Based on the judgment matrix, a fuzzy consistent matrix is further constructed to obtain the subjective weighting vector ${W}_{1}=\{{\omega }_{1}^{1},{\omega }_{1}^{2},\dots ,{\omega }_{1}^{m}\}$.
2. The entropy^30,31^ weighting method is used to weight the indicators. This method calculates the entropy weight of each indicator based on the dispersion of their data using information entropy. The entropy weights are then adjusted based on the contribution of each indicator, resulting in a relatively objective weighting vector ${W}_{2}=\{{\omega }_{2}^{1},{\omega }_{2}^{2},\dots ,{\omega }_{2}^{m}\}$.
3. The composite weights are calculated^32^. Through computation, the weight of a particular evaluation indicator obtained from the FAHP method is denoted as ${W}_{1}$, whereas the weight obtained from the entropy weighting method is denoted as ${W}_{2}$. The composite weight, ${W}_{z}$ , is then determined by combining these two weights:
  1 $${W}_{z}={\alpha }_{1}{W}_{1}^{T}+{\alpha }_{2}{W}_{2}^{T}$$
  where $\alpha$ represents the weighting coefficient. Both the FAHP method and entropy weighting method are equally important in determining the composite weight, hence ${\alpha }_{1}={\alpha }_{2}=0.5$.

### Improved coupling and coordination evaluation model

To depict the synergistic relationship between water resources and social-economy development, an improved coupling coordination degree (CCD) model^33^ is introduced to quantitatively evaluate the coordination and its spatiotemporal variation^34^ in the complex water resources system of Hunan Province. The improved CCD model overcomes the issue of high coupling at low levels by incorporating calculations of the system development index, coupling degree, coordination degree, and coupling coordinative degree. By combining the values of the coupling coordinative degree with the classification criteria of coordination levels, the degree of coupling coordination for each aspect is ultimately determined.

First, the development indices for the WRS and SES should be calculated. The development index is a dimensionless value ranging from 0 to 1, designed to reflect the level of system development. The calculation formulas for the two subsystems are as follows:

2 $$WR\left(x\right)=\sum_{i=1}^{m}{x}_{i}^{*}{\omega }_{i}^{WR}$$

3 $$SE\left(y\right)=\sum_{i=1}^{n}{y}_{i}^{*}{\omega }_{i}^{SE}$$

where $WR\left(x\right)$ represents the development index of the WRS, while $SE\left(y\right)$ represents the development index of the SES. And ${x}_{i}^{*}$, ${y}_{i}^{*}$ denote the standardized values of the respective evaluation indicators within each subsystem. And ${\omega }_{i}^{WR}$, ${\omega }_{i}^{SE}$ represent the weights assigned to each evaluation indicator within the two subsystems. And $m$, $n$ denote the number of evaluation indicators within each subsystem, where, in this case,$m=10$, $n=12.$

Based on the calculation of the development indices, the introduced coupling coordination analysis model is used, and different criteria are set to assess the levels of coupling coordination^35^. The coupling and coordination development relationship between the WRS and SES can be classified into different types, as shown in Table 2.

4 $${CCD}_{h}=\sqrt{{CD}_{h}^{coup}\times {CD}_{h}^{coord}}$$

5 $${CD}_{h}^{coup}=\frac{\frac{1}{\sqrt{2}}\times \frac{WR\left(x\right)+SE\left(y\right)}{\sqrt{{WR\left(x\right)}^{2}+{SE\left(y\right)}^{2}}} -\frac{1}{\sqrt{2}}}{1-1/\sqrt{2}}$$

6 $${CD}_{h}^{coord}=\alpha WR\left(x\right)+\beta SE\left(y\right)$$

where ${CCD}_{h}$ represents the coupling coordinative degree, ${CD}_{h}^{coup}$ represents the coupling degree, ${CD}_{h}^{coord}$ represents the coordination degree, and $\alpha$ , $\beta$ are coefficients that reflect the importance of the subsystems. In this study, we set the coefficient to a certain value to determine its significance, and in this case, $\alpha =\beta =0.5$ .

**Table 2:** Coupling coordinative degree evaluation level criteria.

Indicator	Indicator value	Development stage/development type	
Development index	0.00–0.19	Difference	
	0.20–0.39	Poor	
	0.40–0.59	Fair	
	0.60–0.79	Good	
	0.80–0.99	Excellent	
Coupling coordinative degree	0.00–0.09	Developmental disorders stage	Extremely dysfunctional recession
	0.10–0.19		Severe dysfunctional decline
	0.20–0.29		Moderate dysregulation recession
	0.30–0.39		Mild dysregulation recession
	0.40–0.49	Transition phase	On the verge of dysfunctional decline
	0.50–0.59		Barely disordered recession
	0.60–0.69	Coordinative development stage	Primary coordinative Coupling
	0.70–0.79		Intermediate coordinative coupling
	0.80–0.89		Good coordinative coupling
	0.89–0.99		Excellent coordinative coupling

### Harmonious development evaluation model

**Figure 3:**
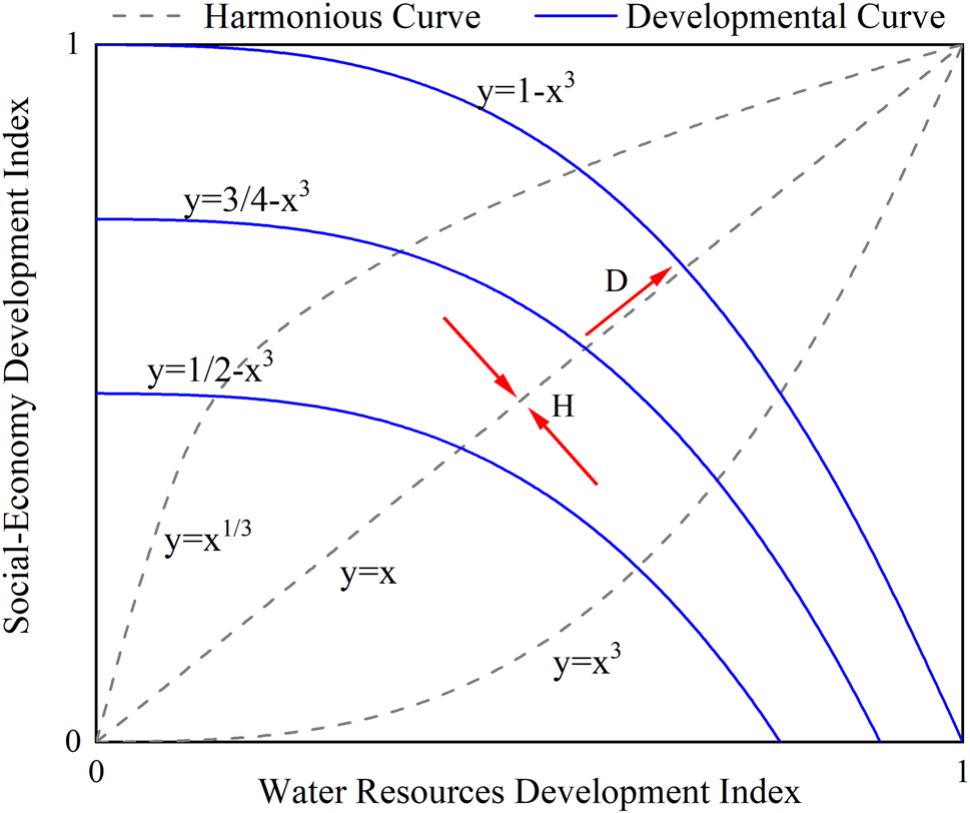
Schematic diagram of harmonious development model.

The harmonious development capacity between the WRS and SES was quantitatively evaluated using the harmonious development evaluation model^36,37^. This model is based on the relationship between the development indices of water resources and social-economy development, and it establishes a rectangular coordinate system in the W-E plane (Fig. 3). The dashed lines $y={x}^{1/3}$, $y=x$, and $y={x}^{3}$ divide the rectangular region into four equally sized parts from the upper-left corner to the lower-right corner. As indicators of harmonious capacity, the directions indicated by the two red arrows in the figure represent a higher level of harmonious capacity as they approach line $y=x$. The solid lines $y=1/2-{x}^{3}$, $y=3/4-{x}^{3}$, and $y=1-{x}^{3}$ divide the rectangular region into four equally sized parts from the lower-left corner to the upper-right corner. As indicators of development capacity, the directions indicated by the red arrows in the figure show that values closer to 1 represent better development capacity. This model can imply the potential harmony and development trends between the WRS and SES.

Based on the connotation of the harmony development model, we calculate the harmony degree H, development degree D, and harmony development degree HD:

7 $$H=\left\{\begin{array}{c}a,a<1\\ 1/a,a>1\end{array}\right.,SE={WR}^{a}$$

8 $$D=SE+{WR}^{3}$$

9 $$HD={D}^{1/2}\times {H}^{1/2}$$

### Improved OECD model

To further illustrate the synergistic relationship between WRS and SES more intuitively, an improved Organization for Economic Co-operation and Development (OECD) model^38^ was introduced to assess the development lag types of the WSE system. The OECD model, based on the changes in the system development index, reflects the relationship between the two subsystems, further enhancing the objectivity and accuracy of the driving factors.

10 $$\upvarepsilon =\frac{\Delta WR}{\Delta SE}$$

where $\Delta WR$ represents the rate of change in the development index of the WRS, and $\Delta SE$ represents the rate of change in the development index of the SES.

Owing to the sustained high economic growth in Hunan Province from 2005 to 2020, the overall level of economic development has shown a continuous upward trend. Based on the above discussions, this study only considers cases where $\Delta SE>0$. The specific descriptions of the corresponding $\upvarepsilon$ types of development lag are provided in Table 3.

**Table 3:** Criteria for the type of development lag of the WSE system.

Positive and negative of $\Delta WR$	Relationship between the size of $\upvarepsilon$	Development lag grade	Development lag type
$\Delta WR$>0	$\upvarepsilon \ge 1$	I	Strong economic and social development lags
	$0.5<\upvarepsilon <1$	II	Weaker water resources, development lags behind
	$\upvarepsilon <0.5$	III	–
$\Delta WR\le 0$	$\upvarepsilon >-0.5$	III	–
	$-1\le\upvarepsilon \le -0.5$	IV	Weaker water resources, development lags behind
	$\upvarepsilon <-1$	V	Strong water resources, development lags behind

When $\upvarepsilon \ge 1$, it indicates a significant lag in social-economy development compared with the improvement in water resources development, which is called a strong social-economy development lag. When $0.5<\upvarepsilon <1$, it indicates that, with economic growth, there is some improvement in water resources, but the former is faster than the latter, resulting in a relatively weak lag in water resources development. When $\upvarepsilon <-1$, it indicates a progressive interaction or a strong lag in water resources development within the WSE system.

**Figure 4:**
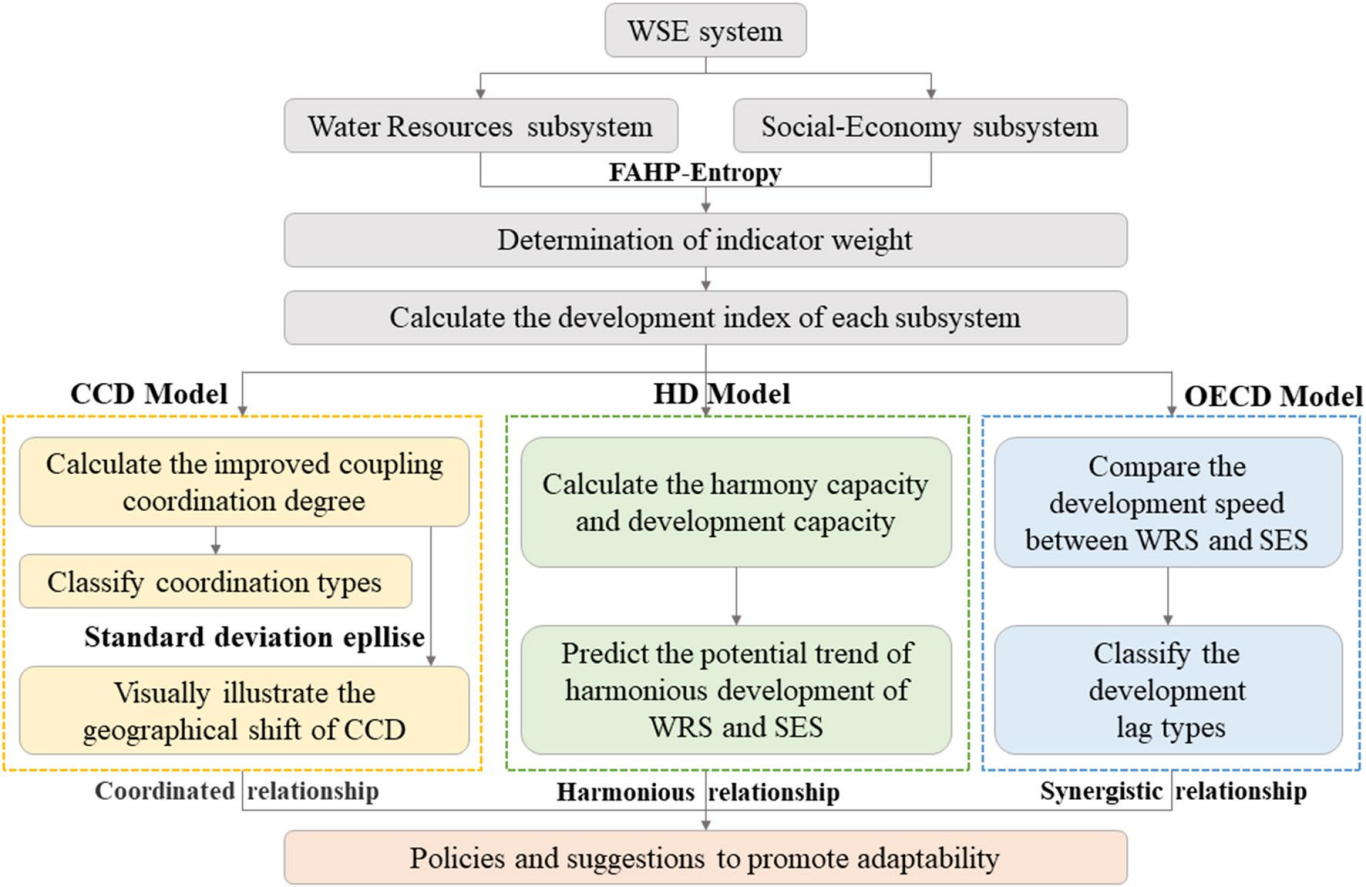
Technical framework.

The technical framework of this study is shown in Fig. 4.

## Results

### Spatiotemporal evolution characteristics of the composite system

#### Temporal evolution characteristics of development index

**Figure 5:**
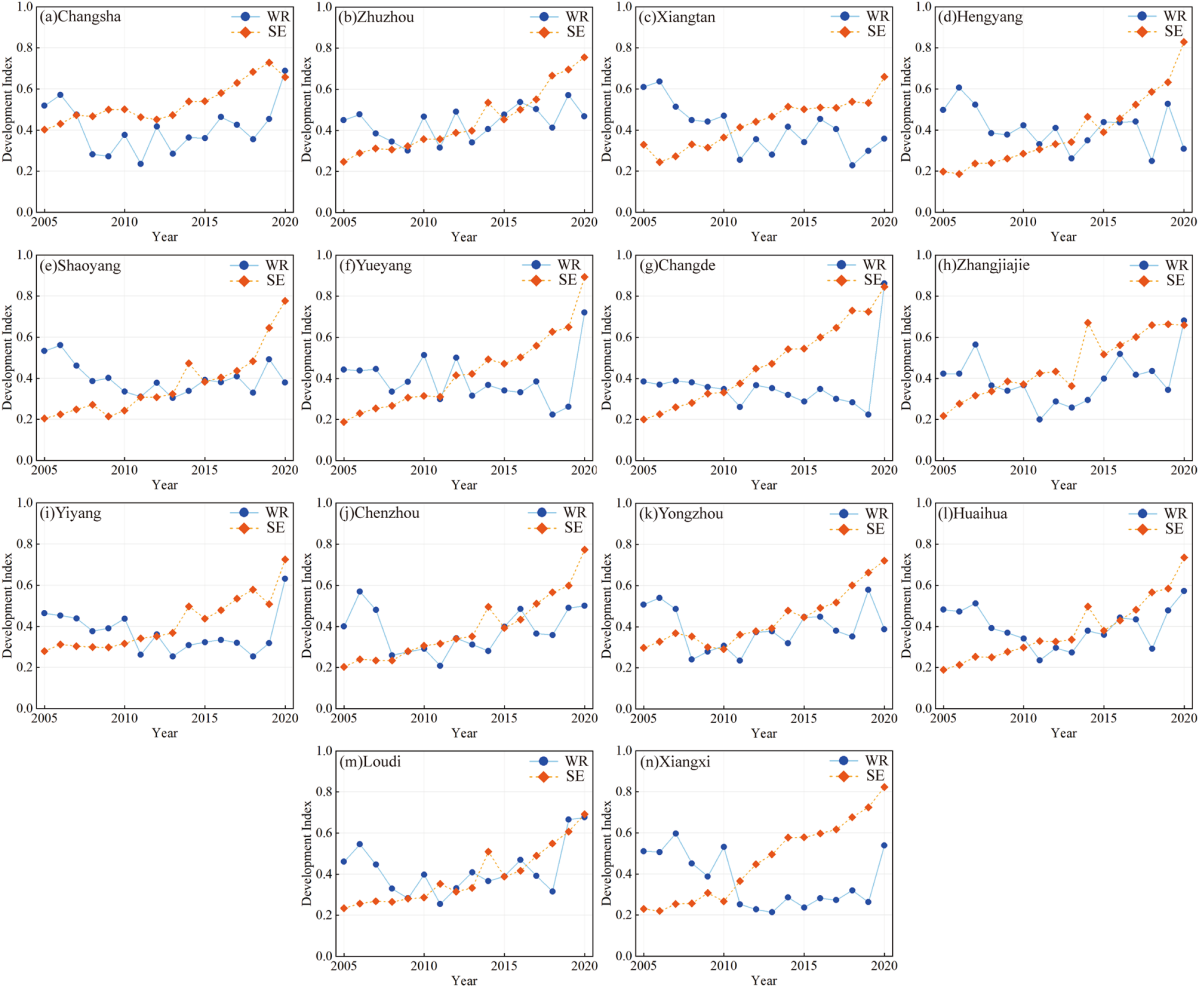
Interannual variation trend of the development index in Hunan Province from 2005 to 2020.

The variation in the development indices for each subsystem of Hunan Province is shown in Fig. 5. When calculating the development index, the combined weights of indicators C1–C10 in the WRS were determined as follows: 0.060, 0.076, 0.069, 0.076, 0.079, 0.135, 0.090, 0.092, 0.231, and 0.091. The combined weights of indicators C11–C22 in the SES were determined as follows: 0.065, 0.063, 0.054, 0.100, 0.107, 0.059, 0.064, 0.132, 0.078, 0.143, 0.065, and 0.070.

The annual variation trend of the WRS development index in Hunan Province from 2005 to 2020 shows significant fluctuations across different cities over 16 years. The variation trend of the WRS in most areas of the province can be divided into three stages: a noticeable decline from 2005 to 2008, fluctuation within the range of 0.2 to 0.6 from 2009 to 2016, and significant changes occurring from 2017 to 2020. The WRS is primarily influenced by local water resource endowment, the level of development and utilization, water consumption structure, and water-saving policies. The implementation of the "Opinions on Implementing the Strictest Water Resource Management System" issued by the State Council in 2012 effectively controlled the degree of water resource development and utilization in Hunan Province, resulting in a decline and fluctuation in the WRS development index in most cities.

From 2005 to 2020, the annual variation trend of the SES development index in Hunan Province showed an overall significant upward trend in most cities. Since 2005, most areas of the province have experienced a continuous upward trend in the SES development index. After reaching the first peak during the years of 2013–2015, the index continued to rise. Apart from Changsha and Zhangjiajie cities, which reached their highest values in 2019 and 2014, respectively, the other cities in Hunan Province reached their peak values in 2020. In 2020, four cities had SES development indices exceeding 0.8, reaching the "excellent" level. For most cities in Hunan Province, the total GDP has increased by about six times between 2005 and 2020, indicating the robust development of secondary and tertiary industries. This has significantly contributed to the rise in the SES development indices across various cities.

#### Spatial evolution characteristics of development index

**Figure 6:**
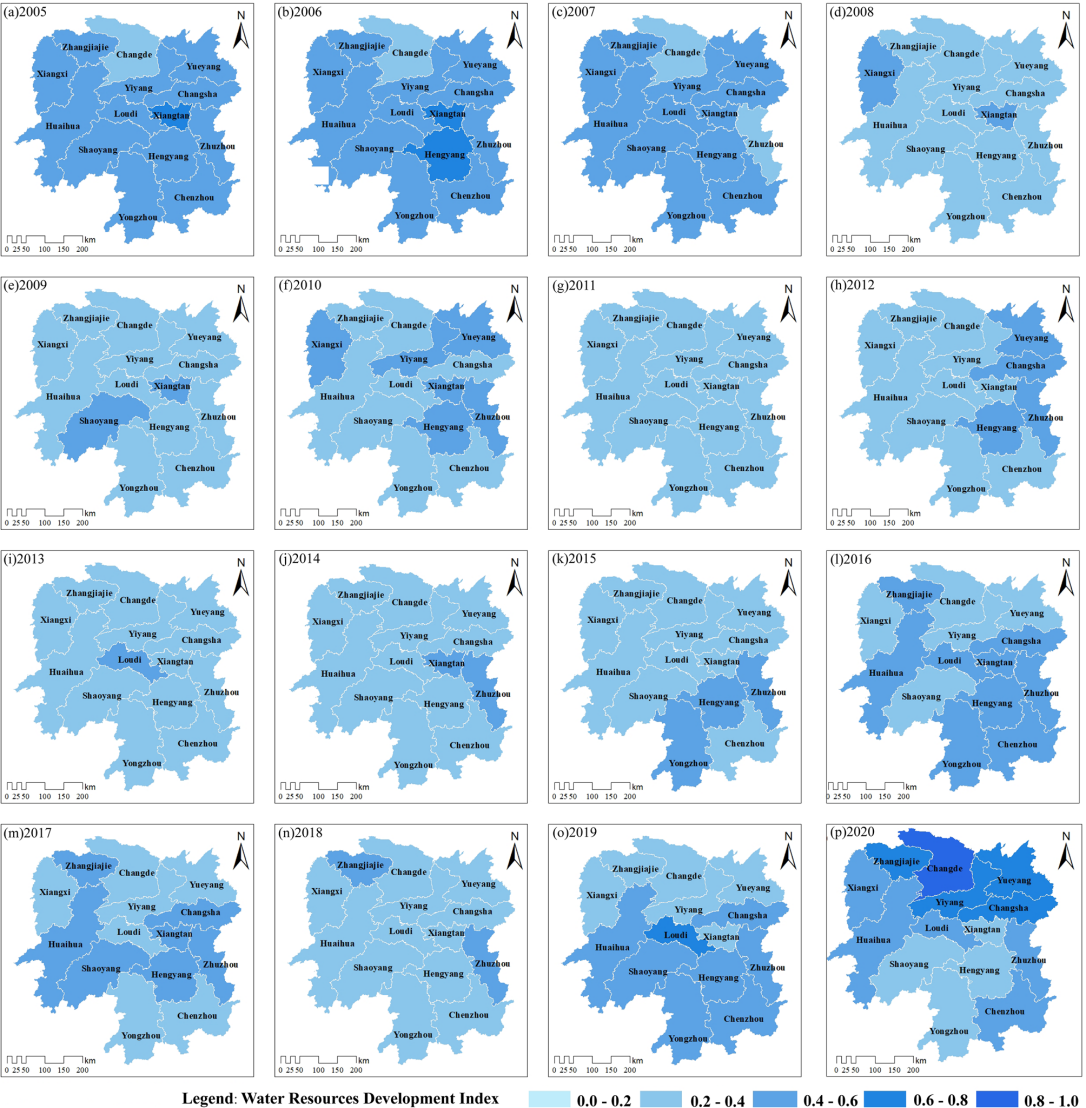
Spatial distribution of the WRS development level in Hunan Province from 2005 to 2020. Graphed by ArcGIS 10.2 and Adobe Illustrator 2021.

According to the spatial evolution of the WRS development level in Hunan Province from 2005 to 2020 (Fig. 6), most areas of Hunan Province exhibited a “fair" or higher level in 2005–2006. From 2007 to 2014, the accelerated industrialization in Hunan Province had a significant adverse impact on the efficient utilization of water resources, resulting in a downgrade of the WRS development level from “fair" to "poor" in most areas of the province. Among them, the minimum precipitation in Hunan Province from 2005 to 2020 occurred in 2011, and during this period, the production methods were relatively extensive. The decrease in precipitation and water pollution issues led to a "poor" water resources development level in all cities of Hunan Province in 2011, representing the lowest level during the period of 2007–2014.

Since 2015, Hunan Province has successively introduced and vigorously implemented a series of water environment restoration policies, including the “Five Major Special Actions for the Comprehensive Treatment of the Dongting Lake Water Environment”, the “Three-Year Action Plan for Ecological and Environmental Restoration”, and the “Eight-Year Plan for Comprehensive Water Environment Governance”. These policies have effectively promoted the overall development of the WRS in the province toward a higher level from 2015 to 2020. In 2018, there was a brief decline in the overall development level in the province, with most cities exhibiting a "poor" level. In 2019, there was some improvement in the southern region of Hunan Province. By 2020, most cities had moved away from the "poor" stage, and it was evident that the WRS development level in the northern part of the province was generally higher than that in the southern part.

**Figure 7:**
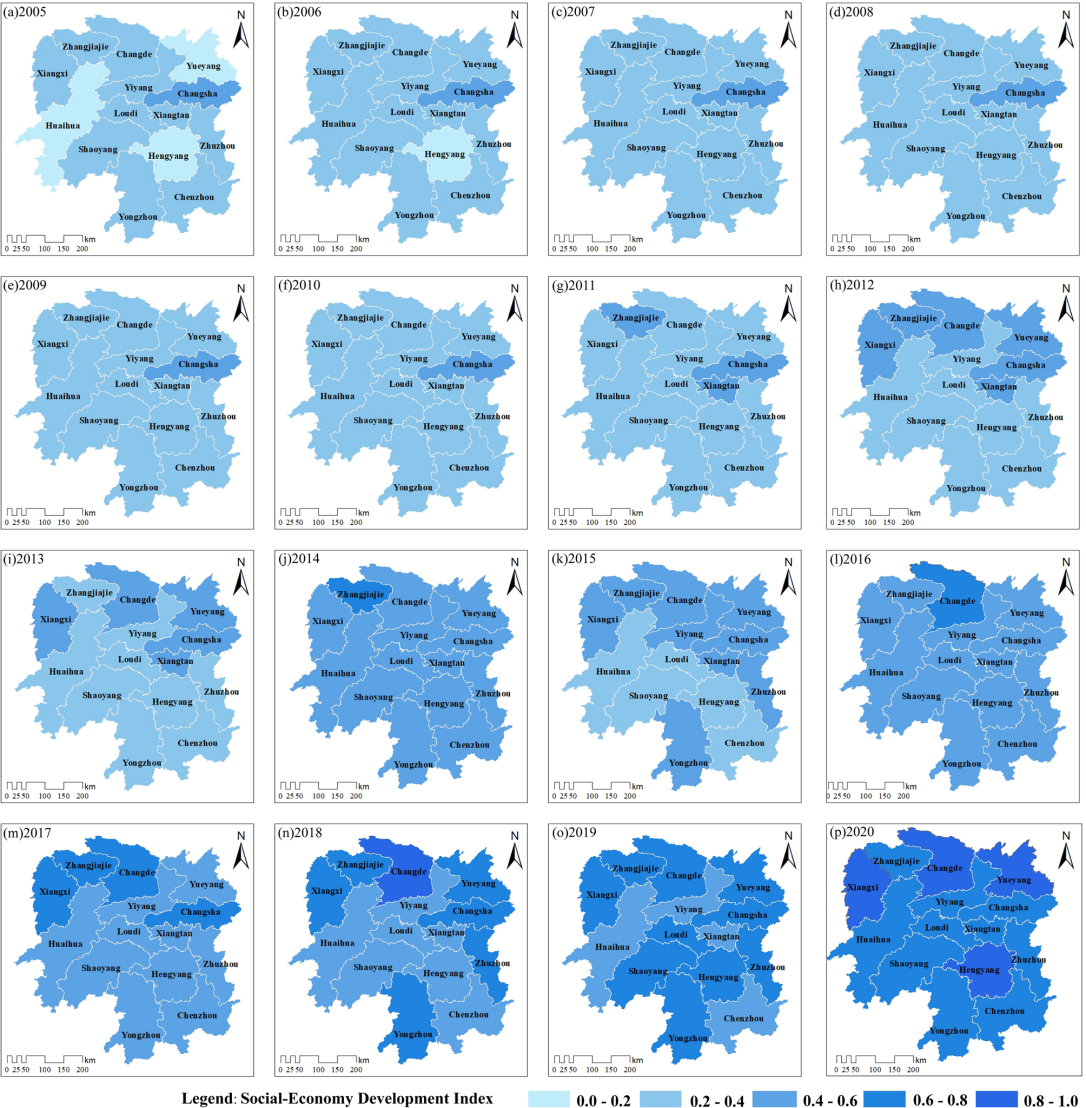
Spatial distribution of the SES development level in Hunan Province from 2005 to 2020. Graphed by ArcGIS 10.2 and Adobe Illustrator 2021.

From the spatial evolution of the SES development level in Hunan Province from 2005 to 2020 (Fig. 7), Huaihua, Yueyang, and Hengyang cities had SES indices below 0.2 in 2005. The overall development status of the province during this period was its lowest between 2005 and 2020. In the years 2006–2007, Huaihua, Yueyang, and Hengyang cities gradually surpassed the 0.2 threshold in their development indices, indicating a slight improvement in their development status from "poor" to "fairly poor." From 2007 to 2010, most cities remained at a "fairly poor" level for an extended period. During the years 2011–2015, the SES development level in Hunan Province exhibited an uneven and unstable pattern between the north and south regions. In 2014, the overall development level of the province reached “fair", showing a brief improvement.

From 2016 to 2020, due to the accelerated industrial upgrading in Hunan Province, the overall SES development level continued to rise, reaching its highest level within this period in 2020. Apart from cities such as Xiangxi, Changde, Yueyang, and Hengyang, which achieved an "excellent" level in 2020, the remaining areas reached a "good" level. Since the 18th National Congress of the Communist Party of China, under the strong leadership of the Party Central Committee with Comrade Xi Jinping at the core, the entire province has vigorously promoted scientific development, providing strong support for rapid social-economy development.

### Spatiotemporal evolution characteristics of the coupling coordinative degree in the composite system

#### Temporal evolution characteristics of the coupling coordinative degree

**Figure 8:**
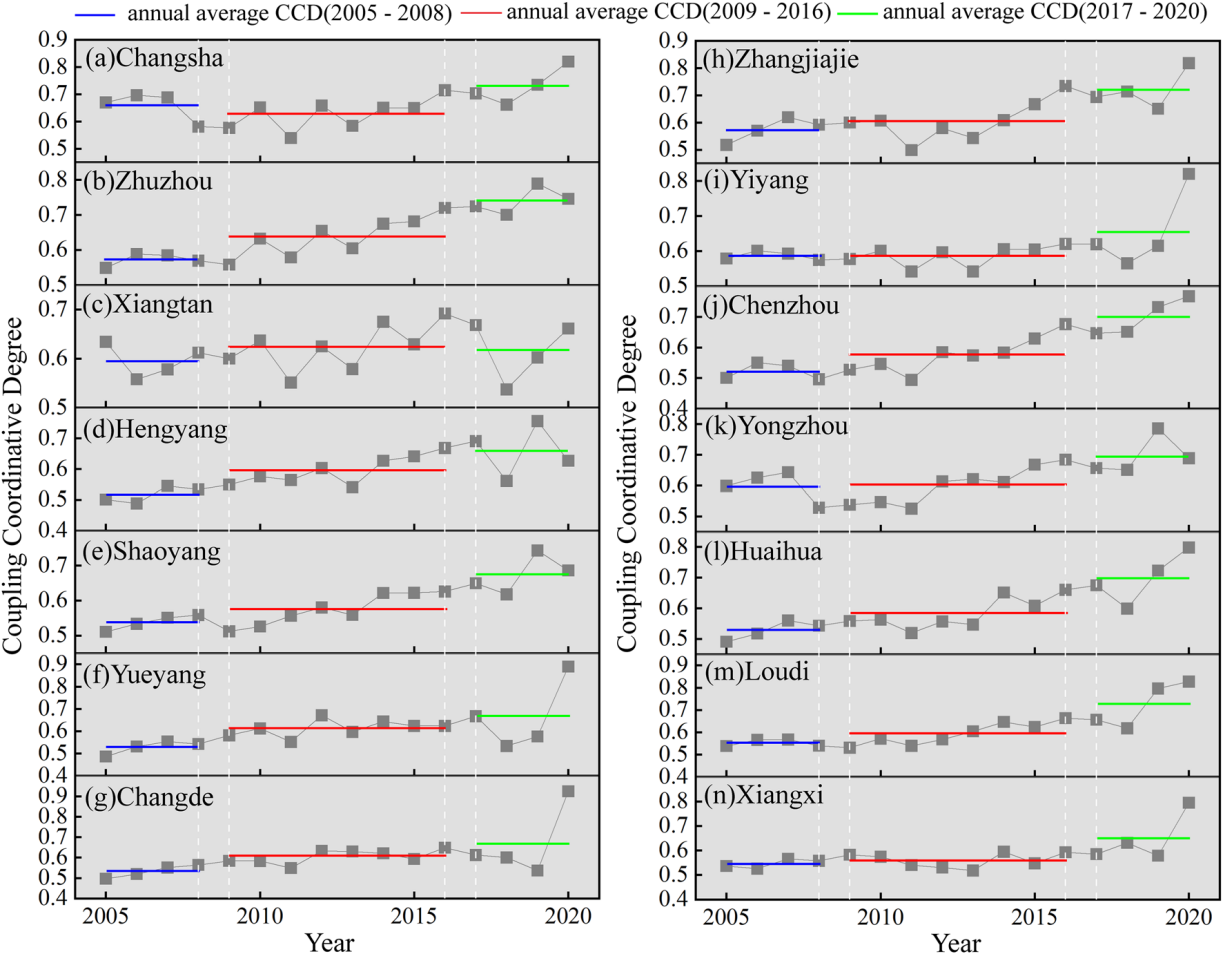
Interannual variation trend of the CCD in Hunan Province from 2005 to 2020.

The trend of the coupling coordinative degree (CCD) in the province was influenced by the development index of water resources and could be broadly divided into three stages: 2005–2008, 2009–2016, and 2017–2020. As shown in Fig. 8, the average CCD in most cities and counties during these three stages showed a gradual improvement.

According to Fig. 8, from 2005 to 2013, most cities and counties in Hunan Province were either in a state of coupling imbalance and decline or fluctuating around the threshold of coordination. In 2013, to implement national energy-saving and emission-reduction policies, Hunan Province actively formulated relevant supporting measures, strictly controlled the development and utilization of water resources, phased out low-end production capacity, and vigorously developed water-efficient and high-value-added electronic information industries. During this period, the closure of a large number of high-water-consumption and low-value-added industries led to a significant reduction in water consumption per 10,000 yuan of GDP and water consumption per 10,000 yuan of industrial value added in the province. However, there is still considerable room for further reducing water consumption per 10,000 yuan of GDP and water consumption per 10,000 yuan of industrial value added in industrially weak cities and counties, such as Xiangxi Prefecture, compared with the national average. The changes described above are visually reflected in the evolution trend of the CCD in Hunan Province. Except for Xiangxi Prefecture, the CCD of other cities exhibited varying degrees of increase after surpassing 0.6 in 2015.

In 2018, there was a significant decline in the CCD of most cities, with several even falling below 0.6. However, the CCD of all cities showed a remarkable upward trend in 2019–2020. In 2020, a total of six cities achieved a CCD above 0.8, with Changde City reaching a state of "excellent coordinative coupling".

#### Spatial evolution characteristics of coupling coordinative degree

**Figure 9:**
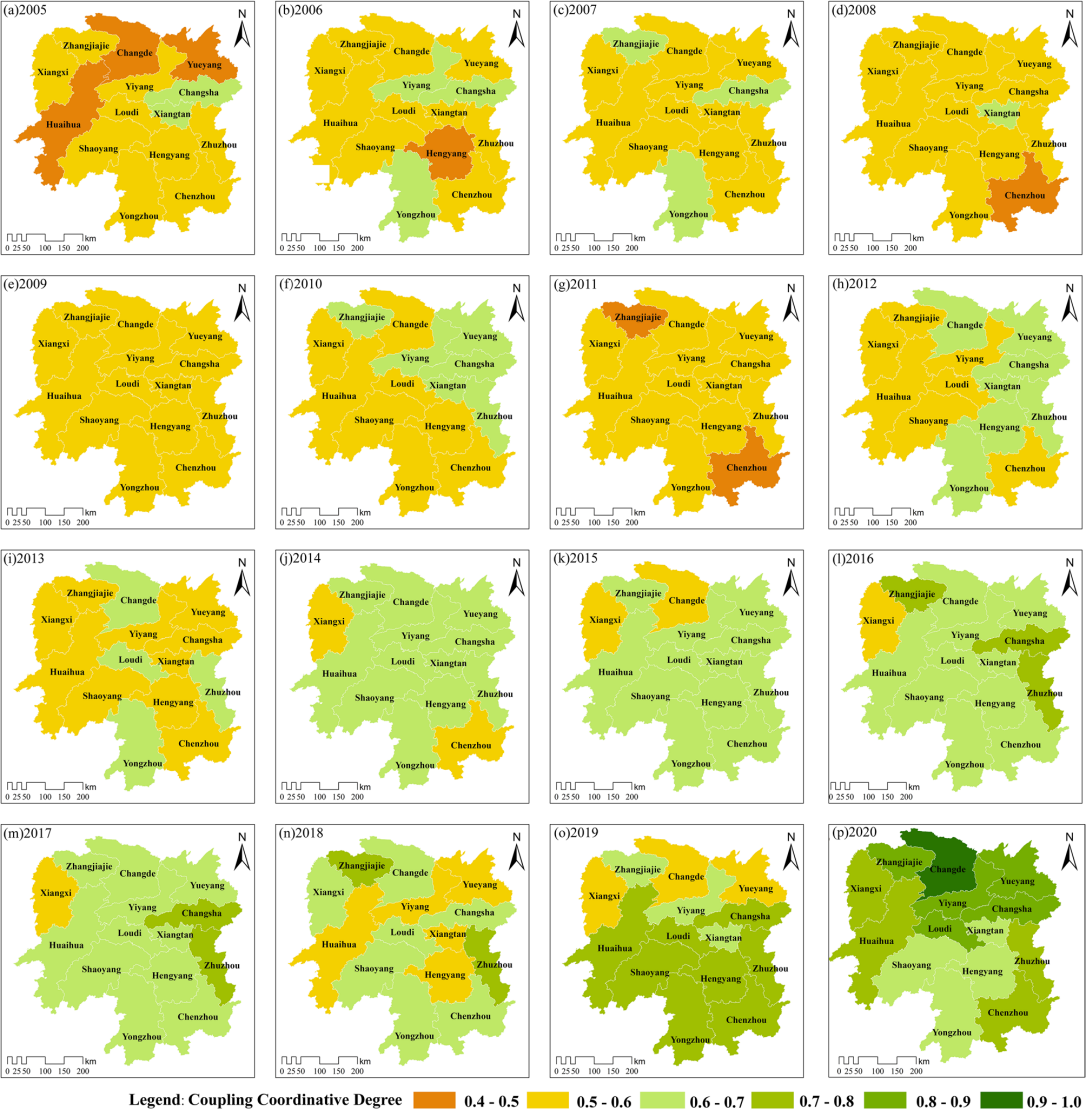
Geographical distribution of the CCD in Hunan Province from 2005 to 2020. Graphed by ArcGIS 10.2 and Adobe Illustrator 2021.

According to Fig. 9, the area in the province exhibiting a "barely coupled and declining" level of coordination decreased in 2005–2006. However, during 2005–2009, most cities in Hunan Province still experienced a state of coupling imbalance and decline, indicating an overall lack of optimistic coordination. During 2010–2013, there was some improvement in the CCD in certain areas. However, the overall coordination and development status in the province remained unstable, with many regions oscillating between the states of coupling imbalance and coordinated coupling.

From 2016 to 2019, the overall CCD in the province exhibited a trend of an "initial increase followed by a decline." In 2019, there was a significant difference in the coordinated development between the northern and southern parts of Hunan Province. Most of the northern regions were classified as "barely imbalanced and declining" or "initial and intermediate coupling coordination", while the southern regions were predominantly in the "intermediate coordinated coupling" level. The level of coordinated development in the northern regions was noticeably lower than that in the southern regions. Until 2020, the coordinated development had a leap across the province, with all cities achieving a state of coordinated coupling.

**Figure 10:**
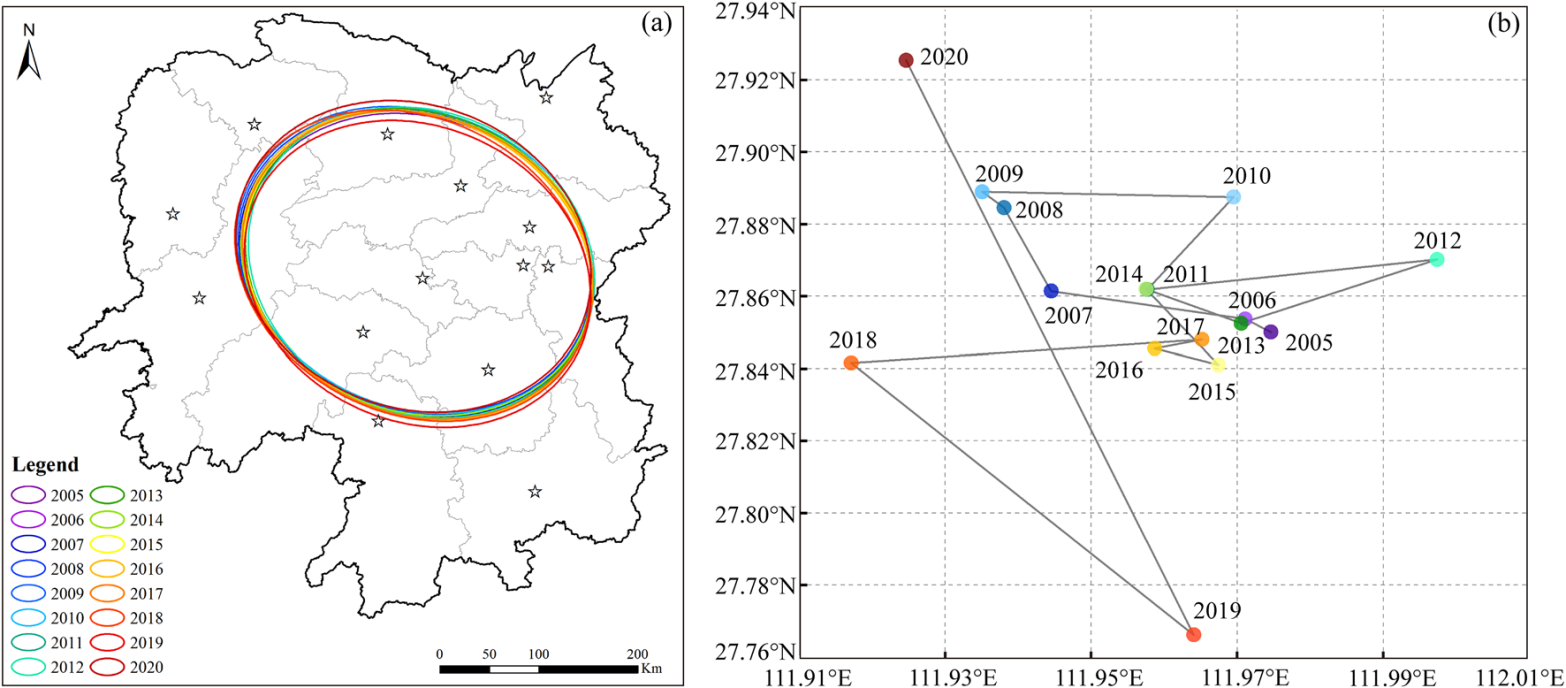
Interannual variation of the standard deviation ellipse in Hunan Province from 2005 to 2020. Graphed by ArcGIS 10.2, Adobe Illustrator 2021 and Origin 2021.

In order to visually illustrate the spatial dispersion of the CCD, this study additionally employed the standard deviation ellipse method^39,40^ to analyze the geographical shift^41^ in the centroid of the CCD from 2005 to 2020. The results are presented in Fig. 10. The figure reveals that the spatial pattern of the standard deviation ellipse in Hunan Province generally follows a northwest-to-southeast direction. Cities such as Yiyang and Loudi, located within the boundaries of the standard deviation ellipse, are the main areas exhibiting coordinated distribution in Hunan province. In 2020, both the length of the major and minor axes had increased compared with 2005, indicating a slight expansion of the ellipse in both the east–west and north–south directions. Additionally, there was a significant increase in the area of the ellipse in 2020 compared with that in 2005, suggesting a larger relative coverage of the ellipse and an increased disparity in the level of coupling and coordination among different cities within the region.

During 2005–2006, the centroid of the ellipse was located near 27.85N, 111.97E. From 2007 to 2009, the centroid gradually shifted towards the northwest. During 2010–2013, the centroid shifted in the direction of "southeast–southwest–northeast–southwest". Between 2013 and 2017, the centroid was mainly concentrated within the range of 27.84N–27.86N and 111.95E–111.97E. From 2018 to 2020, the centroid shifted in the direction of "southwest–southeast–northwest," with a noticeable increase in both the magnitude and trend of movement. In general, the centroid of the coordination phenomenon during 2005–2020 deviated to some extent from the geometric center of the province toward the northeast of Hunan Province. This indicates that the northern region of Hunan Province exhibits a higher level of coordination between water resources and economic-social development compared with the southern region.

### Analysis of harmonious development capacity and development lag types

**Figure 11:**
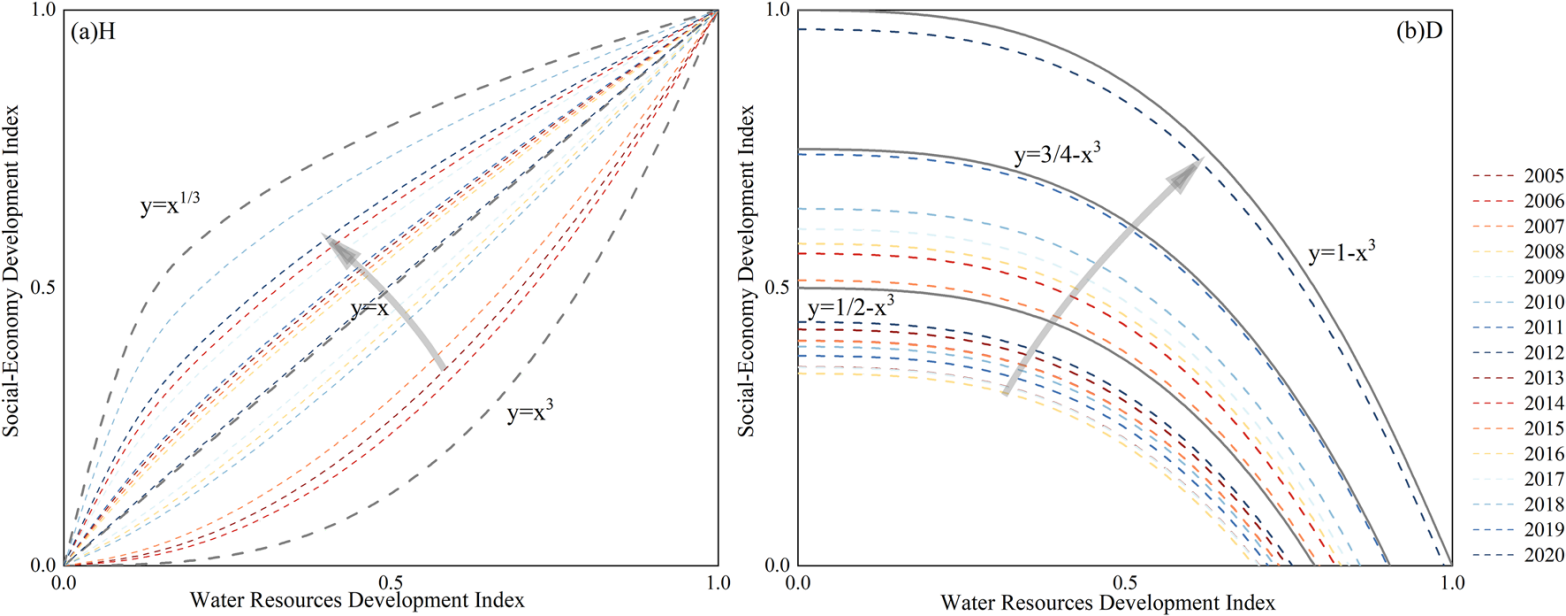
Multi-year changes in the harmonious development capacity in Hunan Province from 2005 to 2020.

Regarding the trend of harmonious development capacity in Hunan Province between 2005 and 2020 (Fig. 11), the position of the harmony degree (H)gradually transitioned from the region bounded by the curves $y={x}^{3}$ and $y=x$ to the region bounded by the curves $y={x}^{3}$ and $y={x}^{1/3}$, during which it approached the dashed line $y=x$ before gradually diverging, indicating an overall upward trend followed by a decline in harmonious development capacity in Hunan Province from 2005 to 2020. Conversely, the development degree (D) in Hunan Province gradually approached the solid line $y=1-{x}^{3}$, indicating a progressive increase in the harmonious development capacity during this period.

Table 4 presents the calculated results of harmonious development capacity and development lag types for each city in Hunan Province from 2005 to 2020. During 2005–2008, except for Changde and Zhangjiajie cities, which were classified as Type III in terms of developmental lag, the remaining 13 cities in Hunan Province were categorized as Type IV or V. This indicated a significant decline in the development index of WRS in most regions during this stage, reflecting an overall pronounced water resource development lag.

**Table 4:** Harmony development capacity and development lag types in Hunan Province from 2005 to 2020.

Cities	2005–2008	Lag types	2009–2016	Lag types	2017–2020	Lag types
	Mean(HD)		Mean(HD)		Mean(HD)	
Changsha	0.641	V	0.597	I	0.662	I
Zhuzhou	0.509	V	0.639	I	0.667	III
Xiangtan	0.477	V	0.598	II	0.553	III
Hengyang	0.395	IV	0.577	II	0.573	II
Shaoyang	0.425	IV	0.560	III	0.605	III
Yueyang	0.434	IV	0.582	IIII	0.546	I
Changde	0.450	III	0.575	III	0.619	I
Zhangjiajie	0.497	III	0.591	I	0.676	I
Yiyang	0.514	V	0.561	III	0.609	I
Chenzhou	0.426	V	0.586	I	0.622	II
Yongzhou	0.528	V	0.618	I	0.627	II
Huaihua	0.412	IV	0.581	II	0.662	I
Loudi	0.456	V	0.586	I	0.757	I
Xiangxi	0.418	IV	0.498	III	0.490	I

From 2009 to 2016, the development lag types in cities such as Changsha, Zhuzhou, Zhangjiajie, Chenzhou, Yongzhou, and Loudi shifted from water lag to economic lag. Specifically, Changsha City experienced a decrease in HD level, indicating a highly uncoordinated situation between WRS and SES. The HD levels among the other five cities showed an increase, indicating the significant acceleration of WRS development, far outpacing SES development. This suggested a more efficient state of water resource utilization and development to a certain extent. On the other hand, cities such as Xiangtan, Hengyang, Yueyang, and Huaihua transitioned to a relatively weak water lag during this stage, with an overall increasing trend in HD levels. This indicated a certain degree of alleviation of the contradiction between social-economy development and water resource scarcity. However, the economic growth rate outpaced the rate of improvement in water poverty conditions, emphasizing that water resource scarcity remains a significant obstacle that cannot be ignored.

From 2017 to 2020, eight cities including Changsha, Yueyang, Changde, Zhangjiajie, Yiyang, Huaihua, Loudi, and Xiangxi, experienced a WRS development rate that far exceeded the pace of SES, indicating a pronounced economic lag. Furthermore, Chenzhou and Yongzhou cities saw an increase in their economic development rates, which surpassed the concurrent rate of water resource development, indicating a relatively weak water resource development lag. In the situation where the cities of Yueyang, Xiangxi, and Hengyang experienced a lack of synchrony between water resources and the rate of social-economy development, there was a pronounced decrease in HD level, necessitating a particular emphasis on enhanced control.

## Discussion

The findings of this paper have some commonalities with previous studies. For example, this paper points out that "the development index of water resources subsystem in Hunan Province shows a decreasing-fluctuating-increasing trend", which is similar to the conclusion of Yang^42^, who used the TOPSIS model to calculate the development index of water resources. The characteristics of water resources development index of various cities calculated in this paper are consistent with the results calculated by Deng^43^, who used the super-efficient EBM model and GML productivity index as well as dynamic panel quantile regression and other measurement methods. Based on the above comparisons, the conclusions of this paper can be considered to be reliable. Compared with the research of spatio-temporal distribution characteristics of Hunan Province completed by Wang^44^, and evolution of coordinated development of social-economy and water resources utilization of Hunan Province calculated by Yang^45^, the indicators used in this paper are richer, reflecting as much as possible the connotations of the appropriateness of water resources and social-economy development from multiple perspectives.

Based on the results, the following suggestions are made for the adaptability development in Hunan Province. Under the premise of maintaining the implementation of water environment improvement policies in Hunan Province, we should actively guide the transformation of highly polluting factories and encourage tertiary industries with low water consumption, high output value, and more friendly to the environment; and prioritize the development of advanced manufacturing industries with higher value-added products. It is also recommended to promote relevant policies, focusing on breaking down the obstacles of administrative divisions between cities and states, deepening industrial cooperation with neighboring cities, and actively improving the sharing of resources and information across the province. Separate zones for regions with acute conflicts between water supply and demand, and construct cross-basin water transfer projects to relief water shortage, and use high-quality water in the ZiShui River basin to improve water quality. The abundant water resources in western Hunan can be transferred to the economically developed regions like Chang-Zhu-Tan urban agglomeration and the Dongting Lake. The high-quality water resources in the Zishui River basin can be used to relief the water shortages in the Heng-Shao-Lou drought corridor and the Xiangjiang River basin, so as to promote the optimal allocation of water resources and the balanced spatial development of water resources and social-economiy development. For cities with low harmonious development capacity, individual control measures should be carried out to accelerate the structural adjustment, upgrade outdated industries, increase investment in fixed assets, and guide the development of independent innovation. It is necesasary to promote industrial water conservation, and gradually enhance the synchronism and harmonious development capacity between WSR and SES. Furthermore, the spatial distribution of industrial layout and water resources should be coordinated and balanced.

## Conclusion

In the study, a comprehensive assessment was conducted from the perspectives of the coordinative relationship, harmonious relationship, and synergistic evolution relationship between water resources utilization and social-economy system among 14 administrative regions of Hunan Province from 2005 to 2020. The conclusions are as follows:

1. The WRS development index in various cities of Hunan Province generally exhibited a declining trend, followed by fluctuations before an upward trend, while the SES development index showed a palpable upward trend.
2. From the perspective of the "Water-Social-Economy" system, Hunan Province overall showed a slight upward trend. Presently, all cities have achieved a state of coordinative coupling, indicating a positive direction of development.
3. The analysis of the evolution of development lag types and harmonious development capacity indicates that the composite "Water-Social-Economy " system in Hunan Province has transitioned from a state of strong water lag in its initial stage, to a state of weak water lag or strong economic lag in the current stage. Simultaneously, the overall harmonious development capacity has shown a trend of an initial increase followed by a decrease. This suggested that the water resources development situation in Hunan Province has improved to some extent.
4. Suggestions for adaptability development were put forward. The transformation and upgrading of industries, construction of water conservancy projects, promotion of water-saving technology could support for the coordinated and sustainable development of “water- social-economy” composite system.

This study can be further deepened as follows: Firstly, the current social-economy and development should be based on the theme of promoting ecological protection and high-quality development. Therefore, the suitability evaluation model should also focus on the competition between water use for living, production, and ecology, so as to avoid crowding out ecological water use in pursuit of development. What’s more, the analysis of the spatial suitability of water resources and social-economy was only carried out for the current situation and historical years. Discussion of the WSE development trend after the completion of the water network in the planning year should be included.

## Data Availability

Publicly available datasets were analyzed in this study. These data can be found in the "Hunan Statistical Yearbook" and the "Hunan Water Resources Bulletin" for 2005–2020. "Hunan Statistical Yearbook" for 2005–2020 can be directly obtained from the People's Government of Hunan Province website http://www.hunan.gov.cn/hnszf/zfsj/tjnj/tygl.html. "Hunan Water Resources Bulletin" for 2005–2020 can be directly obtained from the Hunan Provincial Water Resources Department website http://slt.hunan.gov.cn/slt/xxgk/tjgb/index_3.html.
